# Phlorizin Supplementation Attenuates Obesity, Inflammation, and Hyperglycemia in Diet-Induced Obese Mice Fed a High-Fat Diet

**DOI:** 10.3390/nu8020092

**Published:** 2016-02-16

**Authors:** Su-Kyung Shin, Su-Jung Cho, Un Ju Jung, Ri Ryu, Myung-Sook Choi

**Affiliations:** 1 Department of Physiology & Obesity-related Disease Research Center, Keimyung University School of Medicine, Daegu 702-701, Korea; ssk1210@hanmail.net; 2 Center for Food and Nutritional Genomics Research, Kyungpook National University, 1370 Sankyuk Dong Puk-ku, Daegu 702-701, Korea; chosj1181@naver.com (S.-J.C.); sangsang0119@gmail.com (R.R.); 3 Department of Food Science and Nutrition, Kyungpook National University, 1370 Sankyuk Dong Puk-ku, Daegu 702-701, Korea; 4 Department of Food Science and Nutrition, Pukyong National University, Busan 608-737, Korea; jungunju@naver.com

**Keywords:** phlorizin, obesity, inflammation, insulin resistance, high-fat diet

## Abstract

Obesity, along with its related complications, is a serious health problem worldwide. Many studies reported the anti-diabetic effect of phlorizin, while little is known about its anti-obesity effect. We investigated the beneficial effects of phlorizin on obesity and its complications, including diabetes and inflammation in obese animal. Male C57BL/6J mice were divided into three groups and fed their respective experimental diets for 16 weeks: a normal diet (ND, 5% fat, *w*/*w*), high-fat diet (HFD, 20% fat, *w*/*w*), or HFD supplemented with phlorizin (PH, 0.02%, *w*/*w*). The findings revealed that the PH group had significantly decreased visceral and total white adipose tissue (WAT) weights, and adipocyte size compared to the HFD. Plasma and hepatic lipids profiles also improved in the PH group. The decreased levels of hepatic lipids in PH were associated with decreased activities of enzymes involved in hepatic lipogenesis, cholesterol synthesis and esterification. The PH also suppressed plasma pro-inflammatory adipokines levels such as leptin, adipsin, tumor necrosis factor-α, monocyte chemoattractant protein-1, interferon-γ, and interleukin-6, and prevented HFD-induced collagen accumulation in the liver and WAT. Furthermore, the PH supplementation also decreased plasma glucose, insulin, glucagon, and homeostasis model assessment of insulin resistance levels. In conclusion, phlorizin is beneficial for preventing diet-induced obesity, hepatic steatosis, inflammation, and fibrosis, as well as insulin resistance.

## 1. Introduction

Phlorizin exists in a number of plants, especially in apple trees in which it is abundant. Since its isolation from the bark of the apple tree 180 years ago, it has been studied and used in human medicine [1,2]. Most studies revealed the anti-diabetic effect of phlorizin: phlorizin improved hyperglycemia by blocking renal glucose resorption and intestinal glucose absorption through inhibition of the sodium–glucose symporters [3,4,5,6]. In addition, some studies reported its anti-inflammatory and antioxidant activities [2,3]. However, little is known about the effect of phlorizin on obesity.

Obesity is a serious health problem worldwide; furthermore, obesity-related health costs, complications, and risk factors are staggering [7]. One of the main environmental factors associated with obesity is a high-fat diet (HFD). In rodents (e.g., C57BL/6J mouse), the HFD feeding induced obesity and metabolic diseases similar to the human body [8,9]. Diet-induced obesity in C57BL/6J mice led to leptin resistance [10]. Mice also developed diabetes with increases in plasma glucose, insulin, hepatic triglyceride levels, and changes in the plasma lipid and adipokine levels [11,12]. The adipokines produced and released by white adipose tissue (WAT) are responsible for not only the chronic inflammation but also insulin resistance, particularly in the case of visceral obesity [13]. The goal of the current study was to investigate whether phlorizin has beneficial effects on obesity and its complications, including diabetes and inflammation, by suppressing fat accumulation and regulating plasma glucose, insulin, and adipokine levels in C57BL/6J mice.

## 2. Experimental Section

### 2.1. Animals and Diets

Male C57BL/6J mice were purchased from the Jackson Laboratory (Bar Harbor, ME, USA) at 4 weeks of age. They were individually housed at a constant temperature of 24 °C, with a 12-h light/dark cycle, and fed a pelletized commercial non-purified diet for 1 week after arrival. The mice were then randomly divided into 3 groups (*n* = 10). They were fed the respective experimental diets (based on the AIN-76 semisynthetic diet) for 16 weeks: a normal diet (ND, 5% fat (corn oil), *w*/*w*), high-fat diet (HFD, 20% fat (3% corn oil and 17% lard) plus 1% cholesterol, *w*/*w*) or HFD with phlorizin (PH, 0.02%, *w*/*w*). We mixed all ingredients in the diets and made the diets using a diet mixer. The mice had free access to food and distilled water during the experiment. Their food intake and body weight were recorded daily and weekly, respectively. At the 16th week, the mice were anesthetized with diethyl ether and sacrificed after a 12-h fast. Blood was taken from the inferior vena cava and then centrifuged at 1000× *g* for 15 min at 4 °C. The plasma was separated for analysis of the plasma biomarkers. After blood collection, the liver and adipose tissues were promptly removed, rinsed and weighed. The liver tissues were frozen in liquid nitrogen and stored at −70 °C. This animal study protocol was approved by the Ethics Committee for Animal Studies at Kyungpook National University, Korea (approval No. KNU 2010-4-14).

### 2.2. Plasma Biomarkers and Hepatic Lipids

Plasma-lipid concentrations were determined using commercially available kits. Total-cholesterol, triglyceride, high-density lipoprotein (HDL)-cholesterol (Asan, Seoul, Korea), free fatty acids (FFA; Wako Chemicals, Richmond, VA, USA), apolipoprotein (apo) A-I, and apoB (Eiken, Japan) levels were analyzed.

A multiplex detection kit (Bio-Rad, Hercules, CA, USA) was used to determine the levels of plasma insulin. Additionally, the kit was used to measure the levels of glucagon, leptin, adipsin, adiponectin, tumor necrosis factor (TNF)-α, monocyte chemoattractant protein (MCP)-1, interferon (IFN)-γ, and interleukin (IL)-6. All samples were assayed in duplicate and analyzed with a Luminex 200 Labmap system (Luminex, Austin, TX, USA). Data analyses were performed using the Bio-Plex Manager software version 4.1.1 (Bio-Rad, Hercules, CA, USA). The plasma glucose level was analyzed using a commercially available kit (Asan, Seoul, Korea). The homeostasis model assessment of insulin resistance (HOMA-IR) was calculated as previously described: HOMA-IR = (fasting insulin concentration (mU/L)) × (fasting glucose concentration (mg/dL) × 0.05551)/22.5.

The hepatic lipids were extracted using the method of Folch *et al.* [14], and hepatic lipids levels were analyzed using the same enzymatic kits that were used in the plasma analyses.

### 2.3. Hepatic Enzymes Activities

Enzyme sources were prepared according to the method developed by Hulcher *et al.* [15] with slight modifications. The amount of protein in the enzyme sources was determined using the Bradford [16] method with bovine serum albumin as the standard. Fatty acid synthase (FAS) activity was determined according to the method described by Nepokroeff *et al.* [17]. This involves monitoring the malonyl-CoA-dependent oxidation of NADPH at 340 nm, in which the activity represents the oxidized NADPH nmol·min^−1^·mg of protein^−1^. The phosphatidate phosphohydrolase (PAP) activity was determined using the method of Walton *et al.* [18]. Carnitine palmitoyl transferase (CPT) activity was determined according to the method as described by Markwell *et al.* [19]. Fatty acid β-oxidation activity was measured spectrophotometrically by monitoring the reduction of NAD to NADH in the presence of palmitoyl-CoA, as described by Lazarow [20]. The microsomal 3-hydroxy-3-methylglutaryl-CoA reductase (HMGR) activity was determined as described by Shapiro *et al.* [21]. The microsomal acyl-CoA cholesterol acyltransferase (ACAT) activity was determined according to the method of Gillies *et al.* [22].

### 2.4. Histopathological Analysis

The liver and epididymal fat were removed and fixed in a buffer solution of 10% formalin. Fixed tissues were processed routinely for paraffin embedding, and 4-μm sections were prepared and dyed with hematoxylin-eosin and Masson’s trichrome. Stained areas were viewed using an optical microscope with a magnifying power of 200× or 400×.

### 2.5. Statistical Analysis

The statistical analyses were performed using the statistical package for social sciences software (SPSS, Inc., Chicago, IL, USA). Significant differences between the ND and HFD groups, and between the HFD and the PH groups, were determined using the Student’s *t*-test. Differences were considered to be statistically significant when *p* < 0.05. All data were expressed as the mean and standard error of the mean.

## 3. Results

### 3.1. Body and Fat Weights, Adipocyte Size and Food Intake

**Figure 1:**
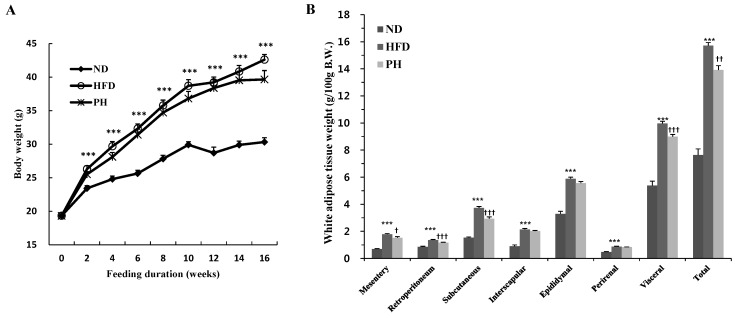

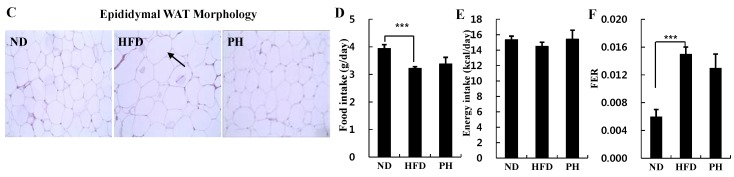
Effects of phlorizin on body and fat weights, adipose tissue morphology and FER: (**A**) body weight; (**B**) white adipose tissue weight; (**C**) epididymal WAT morphology; (**D**) food intake; (**E**) energy intake; and (**F**) FER. C57BL/6J mice (*n* = 10/group) were fed experimental diets for 16 weeks. (**A**,**B**,**D**–**F**) Data are Mean ± Standard Error. Significant differences between HFD *vs.* ND are indicated: *** *p* < 0.001. Significant differences between HFD *vs.* PH are indicated: ^†^ *p* < 0.05; ^††^ *p* < 0.01; ^†††^ *p* < 0.001. (**C**) A representative photomicrograph of the epididymal WAT is shown at 200× magnification; Arrow, adipocytes. ND, normal diet (AIN-76); HFD, high-fat diet (20% fat, 1% cholesterol, *w*/*w*); PH, HFD with 0.02% phlorizin (*w*/*w*). WAT, white adipose tissue; FER, food efficiency ratio.

Body weight significantly increased from the second to the 16th week in the HFD group when compared to the ND group (Figure 1A). Over the same period, PH supplement with HFD tended towards a decrease in body weight, although there was no significant difference (Figure 1A). A HFD significantly increased visceral (mesentery, retroperitoneal, epididymal, and perirenal), subcutaneous and total WAT weights as well as adipocyte size when compared to ND (Figure 1B,C). However, the PH significantly decreased visceral, subcutaneous and total WAT weights and adipocyte size (Figure 1B,C). Food intake was significantly lower in the HFD group as compared to the ND group, while energy intake was not significantly different between the two groups (Figure 1D,E). These led an increase in FER of HFD group (Figure 1F). The PH did not alter food and energy intake and FER.

### 3.2. Plasma Lipids Levels

The HFD group showed significantly higher levels of plasma total cholesterol and triglycerides when compared to the ND group (Table 1). These lipids levels were not affected by PH supplementation, but plasma FFA and non HDL-cholesterol levels increased by HFD were significantly lowered in the PH group compared to the HFD group (Table 1). Moreover, the PH significantly increased plasma apo A-I as well as HDL-cholesterol as compared to the HFD (Table 1). Consequently, the PH significantly increased apo A-I/apo B ratio and HDL-cholesterol/total-cholesterol ratio (HTR), and significantly decreased atherogenic index (AI) compared to the HFD.

**Table 1:** Plasma lipids profiles.

	ND	HFD	PH
Total cholesterol (mmol/L)	2.93 ± 0.12	5.96 ± 0.38 ***	5.51 ± 0.29
Triglycerides (mmol/L)	0.89 ± 0.03	1.48 ± 0.07 ***	1.42 ± 0.06
Free fatty acid (mmol/L)	1.07 ± 0.07	1.29 ± 0.02 *	1.16 ± 0.02 ^††^
HDL-cholesterol (mmol/L)	1.05 ± 0.07	1.99 ± 0.10 ***	2.45 ± 0.12 ^††^
Non HDL-cholesterol (mmol/L)	1.92 ± 0.10	4.01 ± 0.38 ***	3.07 ± 0.21 ^†^
Apolipoprotein-AI (mg/dL)	38.46 ± 0.34	38.59 ± 0.36	48.07 ± 0.51 ^†††^
Apolipoprotein B (mg/dL)	4.47 ± 0.40	9.65 ± 0.53 ***	9.28 ± 0.51
Apo A-I/Apo B	8.97 ± 0.46	4.04 ± 0.18 ***	5.24 ± 0.37 ^†^
HTR (%)	35.69 ± 2.16	32.64 ± 1.24	43.49 ± 1.60 ^†††^
AI	1.75 ± 0.13	2.34 ± 0.21 *	1.21 ± 0.06 ^††^

C57BL/6J mice (*n* = 10/group) were fed experimental diets for 16 weeks. Data are Mean ± Standard Error. Significant differences between HFD *vs.* ND are indicated: * *p* < 0.05; *** *p* < 0.001. Significant differences between HFD *vs.* PH are indicated: ^†^ *p* < 0.05; ^††^ *p* < 0.01; ^†††^ *p* < 0.001. ND, normal diet (AIN-76); HFD, high-fat diet (20% fat, 1% cholesterol, *w*/*w*); PH, HFD with 0.02% phlorizin (*w*/*w*). HTR, (HDL-cholesterol/Total-cholesterol) × 100; AI, atherogenic index: {(Total-cholesterol) − (HDL-cholesterol)}/HDL-cholesterol.

### 3.3. Hepatic Lipids Levels and Enzyme Activity

**Figure 2:**
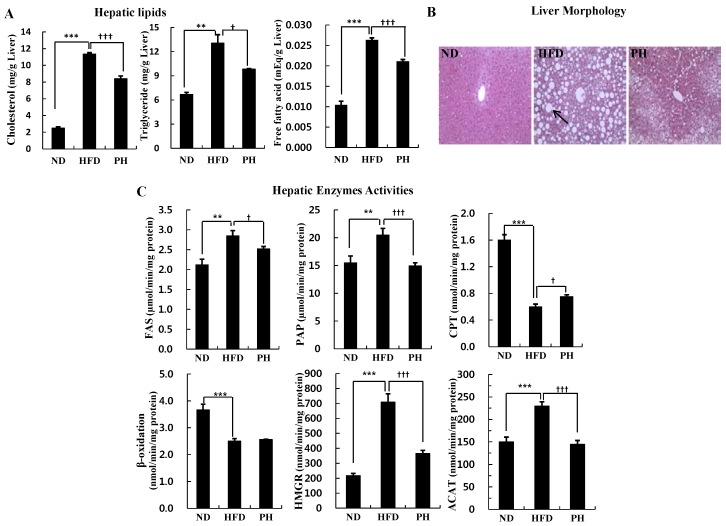
Effects of phlorizin on hepatic lipids (**A**); morphology (**B**); and enzymes activities (**C**) levels. C57BL/6J mice (*n* = 10/group) were fed experimental diets for 16 weeks. (**A**,**C**) Data are Mean ± Standard Error. Significant differences between HFD *vs.* ND are indicated: ** *p* < 0.01; *** *p* < 0.001. Significant differences between HFD *vs.* PH are indicated: ^†^ *p* < 0.05; ^†††^ *p* < 0.001. (**B**) A representative photomicrograph of the liver is shown at 200× magnification. Arrow, lipid droplet. ND, normal diet (AIN-76); HFD, high-fat diet (20% fat, 1% cholesterol, *w*/*w*); PH, HFD with 0.02% phlorizin (*w*/*w*). FAS, fatty acid synthase; PAP, phosphatidate phosphohydrolase; CPT, carnitine palmitoyl transferase; HMGR, 3-hydroxy-3-methylglutaryl-CoA reductase; ACAT, acyl-CoA cholesterol acyltransferase.

The PH significantly decreased hepatic cholesterol, triglyceride and FFA levels that were significantly elevated in the HFD than in the ND (Figure 2A). The PH also decreased the number and size of hepatic lipid droplets that were also increased by the HFD (Figure 2B). The HFD significantly increased the activities of hepatic lipogenic enzymes, FAS and PAP. However, it decreased hepatic CPT activity and β-oxidation as compared to the ND (Figure 2C). Meanwhile, the PH significantly decreased FAS and PAP activities, and increased CPT activity more than the HFD (Figure 2C). The PH also significantly lowered the activities of hepatic lipid biosynthesis and esterification enzymes, HMGR and ACAT, as compared to the HFD (Figure 2C).

### 3.4. Plasma Glycemia Markers

The HFD significantly increased plasma glucose, insulin and glucagon concentrations as well as insulin/glucagon ratio and HOMA-IR value compared to the ND (Table 2). However, the PH significantly decreased these plasma glycemic markers levels (Table 2).

**Table 2:** Plasma glycemia markers.

	ND	HFD	PH
Glucose (mmol/L)	9.71 ± 0.61	15.88 ± 0.41 ***	12.92 ± 0.19 ^†††^
Insulin (ng/mL)	0.41 ± 0.10	1.80 ± 0.21 ***	1.07 ± 0.14 ^†^
Glucagon (pg/mL)	57.04 ± 2.85	95.38 ± 4.30 ***	68.32 ± 5.29 ^††^
Insulin/glucagon	7.28 ± 1.39	18.83 ± 2.75 **	15.39 ± 3.18
HOMA-IR	1.72 ± 0.34	15.07 ± 1.62 ***	5.24 ± 0.57 ^†††^

C57BL/6J mice (*n* = 10/group) were fed experimental diets for 16 weeks. Data are Mean ± Standard Error. Significant differences between HFD *vs.* ND are indicated: ** *p* < 0.01; *** *p* < 0.001. Significant differences between HFD *vs.* PH are indicated: ^†^ *p* < 0.05; ^††^ *p* < 0.01; ^†††^ *p* < 0.001. ND, normal diet (AIN-76); HFD, high-fat diet (20% fat, 1% cholesterol, *w*/*w*); PH, HFD with 0.02% phlorizin (*w*/*w*). HOMA-IR, homeostasis model assessment of insulin resistance.

### 3.5. Plasma Adipokine Levels and Tissue Fibrosis

**Figure 3:**
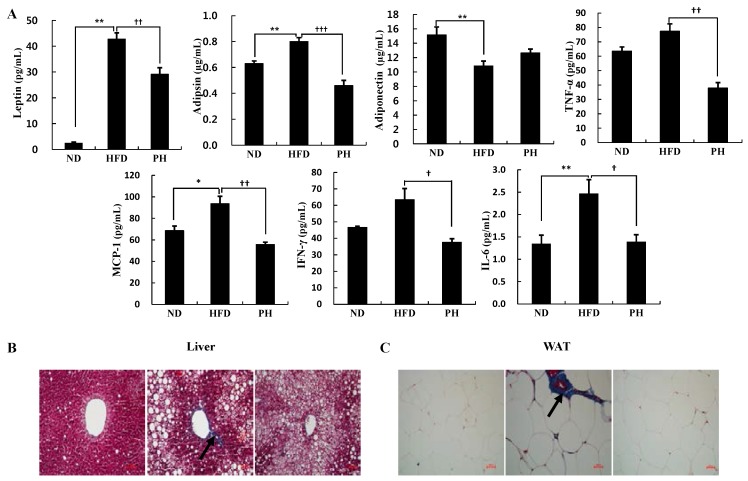
Effects of phlorizin on plasma adipokines (**A**); fibrosis of hepatic (**B**); and adipose tissue (**C**). C57BL/6J mice (*n* = 10/group) were fed experimental diets for 16 weeks. (**A**) Data are Mean ± Standard Error. Significant differences between HFD *vs.* ND are indicated: * *p* < 0.05, ** *p* < 0.01. Significant differences between HFD *vs.* PH are indicated: ^†^ *p* < 0.05, ^††^ *p* < 0.01, ^†††^ *p* < 0.001; (**B**,**C**) Masson’s trichrome stained transverse-section of liver and epididymal fat. Fibrillar collagens, primarily collagen I and III, are stained with blue as indicated with arrowheads. Nuclei and keratin are stained with deep purple and red, respectively. Original magnification (liver, 200×; adipose tissue, 400×). ND, normal diet (AIN-76); HFD, high-fat diet (20% fat, 1% cholesterol, *w*/*w*); PH, HFD with 0.02% phlorizin (*w*/*w*). TNF-α, tumor necrosis factor-alpha; MCP-1, monocyte chemoattractant protein-1; IFN-γ, interferon-gamma; IL-6, interleukin-6; WAT, white adipose tissue.

The HFD significantly elevated plasma leptin and adipsin levels, while it lowered the plasma adiponectin level when compared to the ND (Figure 3A). The PH decreased plasma leptin and adipsin levels with a slight increase in the plasma adiponectin level (Figure 3A). The PH also significantly decreased plasma TNF-α, MCP-1, IFN-γ and IL-6 levels when compared to the HFD (Figure 3A). Hepatic and WAT collagen accumulation was assessed by Masson-trichrome staining, a histological method. The HFD revealed marked increase in collagen accumulation in liver and WAT (Figure 3B,C). The PH appeared to decrease HFD-induced collagen accumulation in liver and WAT (Figure 3B,C).

## 4. Discussion

Phlorizin has been known to improve diabetes from numerous studies [3,4,5,6]. The present study investigated the effects of phlorizin on obesity and related-metabolic diseases in HFD-induced obese mice.

C57BL/6J mice fed a HFD for 16 weeks showed significant increases in body weight, visceral fat weight and adipocyte size when compared to those fed a ND. The HFD group also showed a significantly higher FER level than the ND group, which was consistent with body weight. In previous studies, HFD-induced obese mice exhibited an increased FER, in addition to the deposition of visceral fat and adipocyte hypertrophy [23,24]. We observed that the PH suppressed visceral and subcutaneous fat accumulation and adipocyte hypertrophy in HFD-induced obese mice. However, the FER and body weight were not significantly altered by PH. Both visceral fat and subcutaneous fat can contribute to metabolic disturbances [25]. Furthermore, the PH decreased total WAT weight. Body-fat mass is known to closely correlate with obesity-related metabolic disturbances. These include insulin resistance, impaired insulin secretion, diabetes, hypertension, inflammation and dyslipidemia. Fat stored in adipose tissue is delivered to the liver by the plasma non-esterified fatty acids pool. In the present study, HFD group had higher levels of plasma and hepatic lipids and bigger hepatic lipid droplet than ND group. Meanwhile, PH supplement to HFD decreased the levels of hepatic lipids and plasma FFA as well as hepatic lipid droplet accumulation. The PH also increased plasma HDL-cholesterol and apo A-I, the major apolipoprotein of HDL. The low levels of HDL-cholesterol and apo A-I can occur as part of the metabolic syndrome that includes abdominal obesity, obesity-associated hepatic steatosis, insulin resistance, elevated fasting glucose, and pro-inflammatory states [26].

In the present study, we also found that PH apparently affected the activities of hepatic enzymes, as well as the decrease in WAT weight. The PH decreased the activities of hepatic FAS and PAP, which are key enzymes in the regulation of *de novo* fatty acid and triglyceride synthesis. On the contrary, the CPT activity in hepatic mitochondria was increased by the PH. Mitochondria catalyze the β-oxidation of fatty acids derived from the diet. This pathway constitutes the major process by which fatty acids are oxidized in order to generate energy. CPT allows the initial transport of fatty acids into mitochondria for β-oxidation; therefore, catalyzing the transfer of fatty acids from CoA to carnitine. The PH decreased hepatic cholesterol levels by suppressing activities of HMGR and ACAT that are enzymes involved in hepatic cholesterol metabolism. HMRG, a rate-limiting enzyme in hepatic cholesterol biosynthesis, catalyzes production of mevalonate from HMG-CoA. ACAT is responsible for esterifying free cholesterol to form the cholesteryl ester, which is predominantly stored in liver. Therefore, it seems that the decreased hepatic lipids accumulation in PH group may be attributed to control of hepatic enzymes involved in lipogenesis, cholesterol synthesis and esterification as well as decrease in WAT weight.

Obesity is associated with a state of chronic inflammation, characterized by the abnormal production of pro-inflammatory or anti-inflammatory molecules (so-called “adipokines”). The secretion of these factors from obese adipose tissue provides the evidence for a direct connection between obesity and systemic inflammation [27]. Adipose tissue of obese individuals increases the expression of pro-inflammatory adipokines such as TNF-α, MCP-1, IFN-γ, and IL-6, as well as reduces adiponectin expression [28]. Circulating leptin levels are increased in animals fed a HFD and in animal with inflammation and/or infection states and it affect cytokine production [29]. Leptin directly regulates the production of several cytokines, including TNF-α, IFN-γ, MCP-1 and IL-6 [29]. In the present study, the PH decreased not only leptin levels, but also TNF-α, MCP-1, IFN-γ and IL-6 levels in plasma.

The PH also lowered plasma level of adipsin, which is positively related to adiposity, insulin resistance and dyslipidemia. It plays a role in energy and glucose homeostasis and lipid metabolism [30]. Baas [31] has suggested that adipsin has pro-inflammatory and fibrotic effects in adipose tissue and elsewhere. Not only adipsin but also other adipokines affect inflammation and fibrosis in the liver and WAT [31,32]. For example, plasma leptin, TNF-α and IL-6 levels positively correlate with hepatic injury and fibrosis deposition in WAT and the liver, while adiponectin attenuates hepatic fibrosis [33,34]. Similar to the results in this study, Charlton *et al.* [35] demonstrated that a HFD produced hepatic fibrosis and associated collagen accumulation in C57BL/6 mice. We observed that phlorizin supplement improved HFD-induced collagen accumulation in the liver and WAT.

Since its discovery in 1835, phlorizin has been well known for its anti-diabetic effect [3]. Diet-induced obese mice develop obesity and obesity-induced diabetes showing hyperinsulinemia and insulin resistance, which are related to the abnormal secretion of adipokines [11,12]. In the current study, HFD-induced obese mice displayed elevated circulating glucose, insulin and glucagon levels. The phlorizin supplemented diet ameliorated these responses.

## 5. Conclusions

Our findings suggest that supplementation of phlorizin ameliorates not only insulin resistance, but also obesity in HFD-induced obese mice. The anti-diabetic effect of phlorizin is related to the improvements in hyperglycemia, glucose-regulating hormones levels and the HOMA-IR value. Additionally, the anti-obesity effect of phlorizin is closely connected with its anti-inflammatory effect and decreased adipokine secretion and collagen accumulation. Phlorizin also alleviates nonalcholic fatty liver disease by regulating hepatic lipid-regulating enzymes activities and decreasing collagen accumultaion. Taken together, these findings indicate that phlorizin may be beneficial for preventing diet-induced obesity and inflammation, as well as diabetes. Further studies are needed in order to elucidate the detailed metabolic role of phlorizin.
